# Multiple Power-Saving MSSs Scheduling Methods for IEEE802.16e Broadband Wireless Networks

**DOI:** 10.1155/2014/957158

**Published:** 2014-01-09

**Authors:** Shih-Chang Huang

**Affiliations:** Department Computer Science and Information Engineering, National Formosa University, No. 64, Wénhuà Road, Huwei Township, Yunlin County 632, Taiwan

## Abstract

This work proposes two enhanced multiple mobile subscriber stations (MSSs) power-saving scheduling methods for IEEE802.16e broadband wireless networks. The proposed methods are designed for the Unsolicited Grant Service (UGS) of IEEE802.16e. To reduce the active periods of all power-saving MSSs, the base station (BS) allocates each MSS fewest possible transmission frames to retrieve its data from the BS. The BS interlaces the active periods of each MSS to increase the amount of scheduled MSSs and splits the overflowing transmission frames to maximize the bandwidth utilization. Simulation results reveal that interlacing the active periods of MSSs can increase the number of scheduled MSSs to more than four times of that in the Direct scheduling method. The bandwidth utilization can thus be improved by 60%–70%. Splitting the overflowing transmission frames can improve bandwidth utilization by more than 10% over that achieved using the method of interlacing active periods, with a sacrifice of only 1% of the sleep periods in the interlacing active period method.

## 1. Introduction

The IEEE802.16e [1] provides the service standard for mobile subscriber stations (MSSs) and the base station (BS). This standard specifies two main duplex modes, the frequency division duplex (FDD) mode and the time division duplex (TDD) mode, for communication between the BS and the MSS. The downlink and uplink transmissions in the TDD mode have the same radio band but transfer in different time slots. The downlink transmission, which transfers data from the BS to MSSs, can support point-to-multipoint broadband wireless access. The BS sequentially polls the MSSs and allocates bandwidth to them.

Most MSSs are battery-powered. Prolonging the operation of an MSS requires a well-designed power-saving mechanism. In the communication aspect, an MSS can periodically turn off its transceiver to reduce power consumption and turn it on to retrieve the downlink data from the associated BS. The period during which the MSS turns off the transceiver is called the sleep period. Prolonging the sleep period can save more power but doing so increases the delay time and the overhead of data buffering. An MSS cannot arbitrarily set its sleep period if the quality-of-service (QoS) is considered. The buffered data must be retrieved without violating the constraints of QoS. Therefore, the traffic arrival rate and the constraints of QoS determine the sleep period of an MSS.

Although the IEEE802.16e standard defines three power-saving classes for the connections of MSSs, it does not specify the allocation of sleep periods among multiple MSSs. The schedule methods that are designed to IEEE802.11 [2–5] are unsuitable because their medium access control mechanism differs from IEEE802.16e. The stations in IEEE802.11 compete for the radio medium to transfer data, but the MSSs in IEEE802.16e use Orthogonal Frequency Division Multiplexing (OFDM) frames that are assigned by the BS to deliver data. The OFDM frame is a time slot used by BS to allocate downlink and uplink bandwidth for traffic. Some research has analyzed the power consumption of an MSS [6–8], and some has developed methods for controlling the sleep period of a single MSS [9–12]. Other investigations have been proposed to schedule multiple power-saving MSSs [13–15], but their energy savings can be further improved.

This work presents two methods for scheduling multiple power-saving MSSs, which consider the UGS traffic with strict QoS time constraints. The connections of MSSs operate in the second power-saving class of IEEE802.16e standard. In the first method, the BS interlaces the active periods of MSSs to increase the number of successfully scheduled MSSs. When the interlacing method cannot identify a feasible schedule, the second method splits nonscheduled data to adjacent OFDM frames. This method minimizes the number of OFDM frames for the split data to lessen the additional active time of the corresponding MSS.

The rest of this paper is organized as follows.  Section 2 discusses the related works.  Section 3 introduces the main concept that underlies the two proposed methods.  Section 4 presents the simulation results of the proposed methods, and lastly, Section 5 draws conclusions.

## 2. Related Works

**Figure 1:**
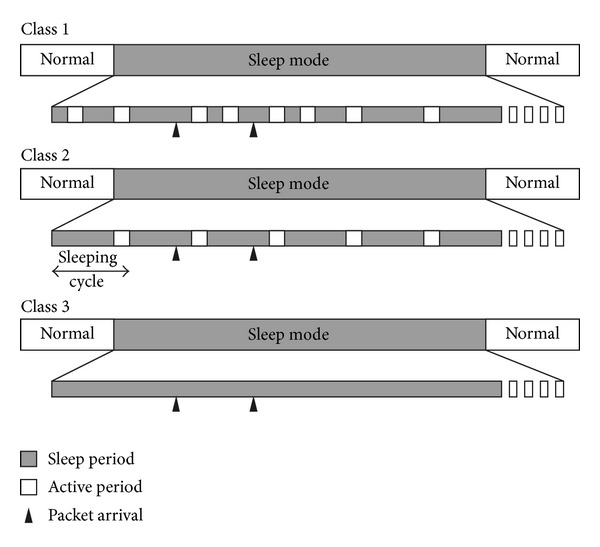
Three power-saving classes of IEEE802.16e.

Figure 1 presents the three power-saving classes of IEEE802.16e. Each MSS negotiates the power-saving parameters (e.g., the sleep period and the active period) with the BS. MSSs retrieve the data that are buffered in the BS in their active periods and turn off their transceivers to save energy during the sleep periods. The durations of both the active periods and the sleep periods are measured in OFDM frames. A *sleep cycle* comprises one sleep period and one active period.

In the first class, the MSS sleeps for a period and then enters the active period, in which the transmission medium is normally monitored. If the MSS does not send or receive data during the active period, then the next sleep period is doubled. This class is suitable for web browsing traffic. In the second class, the durations of a sleep period and an active period are constant. The MSS repeats the sleep period and the active period in a round-robin manner. This class is suitable for the real-time application that periodically sends or receives data, such as voice over IP (VoIP). In the third class, the MSS simply sleeps for a period and then returns to normal operation.

The IEEE802.16e standard does not specify the mechanism for establishing the sleep period of an MSS. Jang et al. utilized the characteristics of all connections in an MSS to establish the sleep period [9]. The connections in this method do not provide QoS support, and this method can be used only for a single MSS. Chen and Tsao aggregated the data that arrived during a sleep period and retrieved them when the MSS was in an active period [10]. So-In et al. tried to minimize the number of bursts on delivering data and minimize the delay jitter [11]. Chang and Lin also proposed a method which considered both type I and the type II power-saving connections in an MSS [12]. Although these three methods considered the connections which have QoS requirements on the time to deliver packets, they can still only be used to schedule a single MSS.

Shi et al. presented a multiple MSSs scheduling method in which the time-constrained connections of the MSSs have the QoS requirements [13]. The MSSs are grouped into a primary MSS and multiple secondary MSSs. Each MSS acts as the primary MSS in turn. The BS only allocates the basic bandwidth to the secondary MSSs to satisfy their QoS constraints, allowing the primary MSS to dominate the remaining bandwidth. The primary MSS aggregates its data and transfers them in one time to shorten the active period. The bandwidth that is allocated to the primary MSS may not suffice as the number of MSS increases. The primary MSS must frequently enter its active period even when the available bandwidth is exhausted.

A simple method for scheduling multiple MSSs is to uniform the duration of the sleep cycles of all MSSs [14]. The scheduling problem can be transformed into the maximum bipartite matching problem and solved using the Ford-Fulkerson maximum flow algorithm. The MSS with the strictest QoS time constraint determines the uniform duration of the sleep cycle. The number of scheduled MSSs in this method is good, but the energy saving is worse. Huang et al. also proposed a multiple MSSs scheduling method, called the power-saving class management scheme (PSS-CAGE) [15]. The duration of the sleep cycle of all MSSs is a power of two. BS aggregates the connections of MSSs to fill as much bandwidth as possible and maximizes the sleep period of the MSSs. When an MSS cannot be scheduled, it generates a sleep cycle with shorter duration and enters the active period more frequently. If the data can be split on demand when an OFDM frame fails to schedule the MSS as required, the MSS can have a longer sleep period.

## 3. Proposed Methods

Consider a network that includes *n* MSSs, each of which has at least one downlink UGS traffic connection. Each packet generated by a connection is under the QoS time constraint and so must be delivered before the time limit is exceeded. The mean traffic interarrival period of any connection is assumed to be shorter than its sleep cycle. In an MSS *S*_*i*_ with *k* connections, the QoS time constraint of the connections governs the maximum duration of the sleep cycle. *S*_*i*_ must switch to the active period to obtain its data before the time limit is exceeded. The sleep cycle of *S*_*i*_, *C*_*i*_, comprises a sleep period and an active period. Let *φ*_*j*_^*i*^ be the QoS time constraint of connection *j* in *S*_*i*_. The *C*_*i*_ and *φ*_*j*_^*i*^ are measured by the OFDM frame. Since *C*_*i*_ must be limited by the connection with a minimum QoS time constraint, it can be represented as

(1) $$\begin{array}{c} C_{i}=\min\left(\varphi_{1}^{i},\varphi_{2}^{i},\varphi_{3}^{i},\ldots ,\varphi_{k}^{i}\right). \end{array}$$

BS allocates enough OFDM frames for *S*_*i*_ after it has determined the duration of the sleep cycle. Let *τ*_*i*_ be the mean traffic generation rate of *S*_*i*_ in an OFDM frame and let *Ω* be the downlink bandwidth capacity of an OFDM frame. The minimum number of OFDM frames that can provide enough bandwidth for the data that are generated within *C*_*i*_, *ω*_*i*_, is given in terms of *C*_*i*_, *τ*_*i*_, and *Ω*. Consider the following:

(2) $$\begin{array}{c} \omega_{i}\geq \frac{\tau_{i}\times C_{i}}{\omega_{i}}. \end{array}$$

Next, the BS schedules the data of *S*_*i*_. The proposed methods are elaborated in the next two subsections.

### 3.1. Interlacing Active Periods (IAP) Method

**Figure 2:**
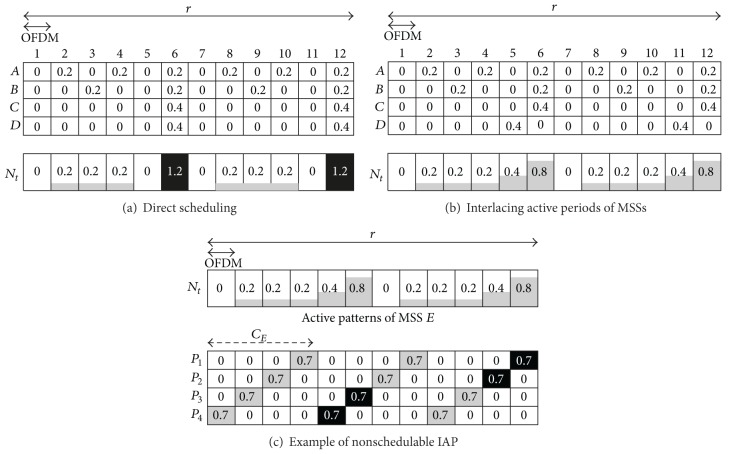
Scheduled active OFDM frames of multiple MSSs.

The first method for maximizing the bandwidth utilization interlaces the downlink OFDM frames of the MSSs. It is called the Interlacing Active Periods (IAP) method. The OFDM frames that are allocated for an MSS to download data are called the active OFDM frames.  Figure 2 presents the underlying concept. Consider a network with four MSSs: *A*, *B*, *C*, and *D*. The durations of their sleep cycles are two, three, six, and six OFDM frames. Their *ω*_*i*_ | {*i* = *A*, *B*, *C*, *D*} are 20%, 20%, 40%, and 40% of the capacity of an OFDM frame. The scheduled order of these four MSSs is *A*, *B*, *C*, and *D*. In Figure 2, *N*_*t*_ is a one-dimensional array which represents the consumed bandwidth of each OFDM frame in a sleep cycle. *N*_*t*_(*x*) is the consumed bandwidth of the *x*th OFDM frame. *N*_*t*_(*x*) ≥ 1 implies that the bandwidth of the *x*th OFDM frame has been completely used.

In Figure 2(a), MSSs are scheduled directly without interlacing of their allocated downlink OFDM frames. MSS *D* cannot be scheduled because the sixth and the 12th OFDM frames overflow when its data are added. However, the BS can schedule MSS *D* if its active OFDM frames can be shifted one OFDM frame to left. The result is shown as Figure 2(b). In the following, a schedule that can potentially place the active OFDM frames of an MSS is called an *active pattern*.

In the example in Figure 2(b), the sequence pattern *N*_*t*_(1), *N*_*t*_(2),…, *N*_*t*_(6) repeats every six OFDM frames. The length of this repeating sequence, *r*, is the lowest common multiple (lcm) of the durations of the sleep cycles of all scheduled MSSs. Therefore, the BS has only to check a segment with length *r* on *N*_*t*_(*x*) to identify the active pattern for a newly joined MSS. This sequence pattern is represented as a matrix *X*, *X* ∈ *ℛ* ^1×*r*^.

Let the sleep cycle of the newly joined MSS *S*_*a*_ be *C*_*a*_ and the bandwidth for delivering the data generated within the period *C*_*a*_ is *ω*_*a*_. The possible active patterns can be represented as a matrix *Y* ∈ *ℛ* ^*C*_*a*_×*C*_*a*_^. Each row in the matrix *Y* represents one possible active pattern, and each element in a row is the required bandwidth of the corresponding OFDM frame. The BS computes the *r*′ = lcm{*C*_*a*_, *r*} and reformats *X* as *X*′ ∈ *ℛ* ^1×*r*′^ and *Y* as *Y*′ ∈ *ℛ* ^*C*_*a*_×*r*′^, which are given by

(3) $$\begin{array}{c} X^{\prime }=\left\{X_{1},X_{2},X_{3},\ldots ,X_{m}\right\},\;\forall X_{i\;|\;i=1,2,3,\ldots ,m}=X,\\ Y^{\prime }=\left\{Y_{1},Y_{2},Y_{3},\ldots ,Y_{n}\right\},\;\forall Y_{i\;|\;i=1,2,3,\ldots ,n}=Y. \end{array}$$

The *m* and *n* in (3) are constants such that *r* × *m* = *C*_*a*_ × *n* = *r*′. Accordingly, the *N*_*t*_ with length *r*′ that is generated by each active pattern of *S*_*a*_ can be represented as rows in the matrix *Z* ∈ *ℛ* ^*C*_*a*_×*r*′^. The elements of *Z* can be computed by

(4) $$\begin{array}{c} Z_{i,j}=\sum_{i=1}^{C_{a}}\sum_{j=1}^{r^{\prime }}\left({X_{1,j}^{\prime }}_{}+{Y_{i,j}^{\prime }}_{}\right). \end{array}$$

Hence, (5) yields the maximal element in the *k*th row of *Z*. Consider the following:

(5) $$\begin{array}{c} I_{k}=\overset{r^{\prime }}{\underset{j=1}{\max}}\left(Z_{k,j}\;|\;Y_{k,j}\neq 0\right). \end{array}$$

Finally, the BS uses the first active pattern with minimal *I*, P*, to schedule the *S*_*a*_ as in

(6) $$\begin{array}{c} P^{*}=\min\left(I_{1},I_{2},\ldots ,I_{C_{a}}\right). \end{array}$$

The BS can schedule *S*_*a*_ if all elements in P* are less than one; otherwise, the BS notifies *S*_*a*_ of insufficient bandwidth.

The time complexity of the IAP method depends on the sum of matrices *X* and *Y* and is *O*(*C*_*a*_ × *r*′); that of the procedure to find the P* is also *O*(*C*_*a*_ × *r*′). Since *r*′ = *C*_*a*_ × *n*, the time complexity of the IAP method is 2 × *O*(*C*_*a*_^2^ × *n*) ∈ *O*(*C*_*a*_^2^).

The following example illustrates the steps for finding the P* of the example in Figure 2(b). *X* = [0.4,0.2,0.2,0.2,0, 0.8] after MSS *C* is scheduled, and the corresponding matrices *Y* and *Z* are as given by

(7) $$\begin{array}{c} Y=\left[\begin{array}{cccccc} 0 & 0 & 0 & 0 & 0 & 0.4\\ 0 & 0 & 0 & 0 & 0.4 & 0\\ 0 & 0 & 0 & 0.4 & 0 & 0\\ 0 & 0 & 0.4 & 0 & 0 & 0\\ 0 & 0.4 & 0 & 0 & 0 & 0\\ 0.4 & 0 & 0 & 0 & 0 & 0 \end{array}\right], \end{array}$$

(8) $$\begin{array}{c} Z=\left[\begin{array}{cccccc} 0.4 & 0.2 & 0.2 & 0.2 & 0 & 1.2\\ 0.4 & 0.2 & 0.2 & 0.2 & 0.4 & 0.8\\ 0.4 & 0.2 & 0.2 & 0.8 & 0 & 0.8\\ 0.4 & 0.2 & 0.8 & 0.2 & 0 & 0.8\\ 0.4 & 0.8 & 0.2 & 0.2 & 0 & 0.8\\ 0.8 & 0.2 & 0.2 & 0.2 & 0 & 0.8 \end{array}\right]. \end{array}$$

In this example, P* = (0,0, 0,0, 0.4,0) is the second row in *Z* and *I*_2_ = 0.8. BS can schedule MSS *D* because all elements in P* are less than one.

However, the IAP method may fail to find a feasible schedule for an MSS, even if enough bandwidth remains. As Figure 2(c) shows, MSS *E* whose *C*_*E*_ = 4 and *ω*_*E*_ = 0.7 joins after MSS *D*. The active pattern P* is *P*_2_. Although the accumulated free bandwidth in the sleep cycle exceeds that required for MSS *E*, the BS cannot use the IAP method to schedule the MSS *E* because adding P* to *N*_*t*_ yields *N*_*t*_(11) = 1.1 > 1. The 11th OFDM frame is called the *overflowing OFDM frame*.

### 3.2. Splitting Overflowing Frame (SOF) Method

In the example of Figure 2(c), the BS must split the data in the overflowing OFDM frames to schedule MSS *E*. The cost to MSS *E* is the additional active OFDM frames. This process is the core of the splitting overflowing frame (SOF) method. In the first step of SOF, the BS identifies the overflowing OFDM frames of each active pattern of MSS *E*. Let *e*_*k*,*j*_ designate the overflowed state of *N*_*t*_(*j*) after the *k*th active pattern has been added to *N*_*t*_. It is defined as

(9) $$\begin{array}{c} e_{k,j}=\{\begin{array}{cc} 1, & \text{if}\;\;Z_{k,j}>1,\\ 0, & \text{otherwise}. \end{array} \end{array}$$

Assume that *O*_*k*_^*E*^ is the total number of overflowing OFDM frames that are generated by the *k*th active pattern. It is defined as

(10) $$\begin{array}{c} O_{k}^{E}=\sum_{j=1}^{r^{\prime }}e_{k,j}\;|\;k=1,2,3,\ldots ,C_{E}. \end{array}$$

**Figure 3:**
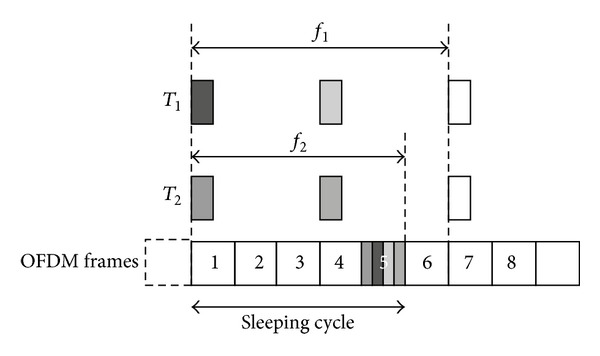
Split data in active OFDM frame.

The transmission of split data can be postponed before the QoS time constraint is violated. In the example in Figure 3, the MSS has two connections *C*_1_ and *C*_2_, whose QoS time constraints are *φ*_1_ = 6 and *φ*_2_ = 5. The sleep cycle of this MSS is five OFDM frames long, and the fifth OFDM frame is the active OFDM frame. All arriving data within the sleep cycle are aggregated in the active OFDM frame for transmission. The first packet of *C*_2_ must be transferred before the fifth OFDM frame to prevent violation of the QoS time constraint. The packets in the active OFDM frames except for the first packet of connection *C*_2_ can be transmitted in the sixth OFDM frame without violating the QoS time constraint.

Let Δ_*k*,*j*_ represent the amount of data, which exceeds the capacity of *N*_*t*_(*j*) that uses the *k*th active pattern, and let Θ_*k*,*j*_ be the data of the *j*th OFDM frame in the *k*th active pattern that cannot be split owing to the QoS time constraint. To meet the QoS time constraint, let the earliest expiration time of the data in Δ_*k*,*j*_ be *q* OFDM frames after the *j*th OFDM frame. The *k*th active pattern has a feasible schedule if (11) is satisfied for all *e*_*k*,*j*_ = 1. Consider the following:

(11) $$\begin{array}{c} \left(X_{j}+\Theta_{k,j}\right)\leq \Omega ,\;\;\overset{q}{\underset{i=j}{\max}}\left(\Omega -X_{i}\right)>\Delta_{k,j}. \end{array}$$

Consequently, one split may not yield a feasible schedule. Data can be split more times to increase the probability of successful scheduling. The cost is an increase in the number of active OFDM frames. If one splitting is carried out, then the minimal number of additional active OFDM frames, min_*k*=1_^*C*_*E*_^(*O*_*k*_^*E*^), that is introduced by the active pattern can be obtained if (12) is satisfied for all *e*_*k*,*j*_ = 1 with *j* = 1,2, 3,…, *r*′. Consider the following:

(12) $$\begin{array}{c} \left(X_{j}+\Theta_{k,j}\right)\leq \Omega ,\;\;\left(\left(\Omega -X_{i}\right)>\Delta_{k,j}\right). \end{array}$$

In the example of Figure 2(c), the SOF method will select the modified active pattern *Z*_2_* = [0,0, 0.7,0, 0,0, 0.7,0, 0,0, 0.6,0.1] to schedule MSS *E*. The algorithm of SOF is shown in Algorithm 1.

## 4. Simulation Results

This section evaluates the proposed IAP and SOF methods. The PSS-CAGE [15] and the Direct methods, whose BS notifies an MSS as soon as its traffic arrives, are considered for comparison. The simulation is implemented with a C++ program. The evaluation metrics includes the average number of scheduled MSSs, the average sleep ratio of an MSS, and the bandwidth utilization. The sleep ratio is the duration of sleep periods over all simulation durations.

### 4.1. Setup of Environment

The simulation considers the second power-saving class as defined in IEEE802.16e. The parameters of the voice connections in the simulation are those recommended by the International Telecommunication Union (ITU) Telecommunication Standardization Sector (ITU-T). Each MSS has five operational UGS voice connections. The traffic interarrival rate for these voice connections is either 20 ms or 40 ms. The QoS delay constraint of a connection is selected from {150 ms, 200 ms, 250 ms, and 300 ms}. The packet size is 100 bytes. The BS can offer a maximal bandwidth capacity to all of the MSSs of 16 Mbps. The duration of an OFDM frame is 5 ms. The simulation is carried out until BS fails to schedule a join MSS. Simulation results are obtained from 300 different runs.

### 4.2. Numerical Results

**Figure 4:**
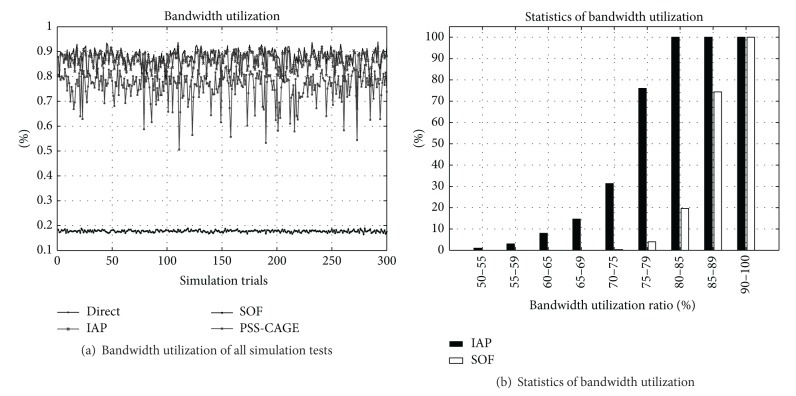
Bandwidth utilization.

Figure 4(a) presents the bandwidth utilization. Without an effective scheduling strategy, the active OFDM frames of the MSSs severely overlap each other in the Direct method. Therefore, the bandwidth utilization achieved using the Direct method is less than 20%. IAP method can achieve 50% to 85% bandwidth utilizations by interlacing the active OFDM frames. The PSS-CAGE and SOF can achieve higher bandwidth utilization by rescheduling the active OFDM frames of the MSSs. Their bandwidth utilizations are very close to each other.

Figure 4(b) presents the statistic bandwidth utilization of the IAP and the SOF methods. More than 80% of the tests yield a bandwidth utilization of more than 70%. The bandwidth utilization that is achieved using the SOF method is 70%–95%. Approximately 95% of tests yield a bandwidth utilization of more than 80%. These results reveal that bandwidth utilization can be improved by splitting the overflowing OFDM frames.

**Figure 5:**
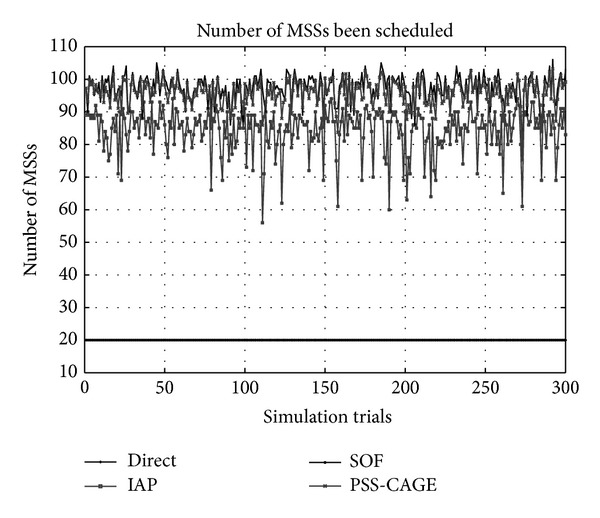
Number of scheduled MSSs.

Figure 5 plots the number of MSSs that are successfully scheduled by the BS. The evaluation is carried out by scheduling the MSSs one at a time to the network until the first one fails to be scheduled. The overlapping of active OFDM frames between MSSs causes the number of MSSs that are scheduled by the Direct method to be under 20. By interlacing the active OFDM frames of the MSSs, the IAP method can avoid most overlaps of the MSSs. Therefore, the number of scheduled MSSs is close to four times that achieved using the Direct method. Scattering the aggregated data to additional active OFDM frames makes the numbers of MSSs that are scheduled by SOF and PSS-CAGE very close to each other. These methods can schedule more than 10% MSSs than the IAP method. The number of scheduled MSSs is also related to the results concerning bandwidth utilization. Increasing the number of MSSs increases the bandwidth utilization.

**Figure 6:**
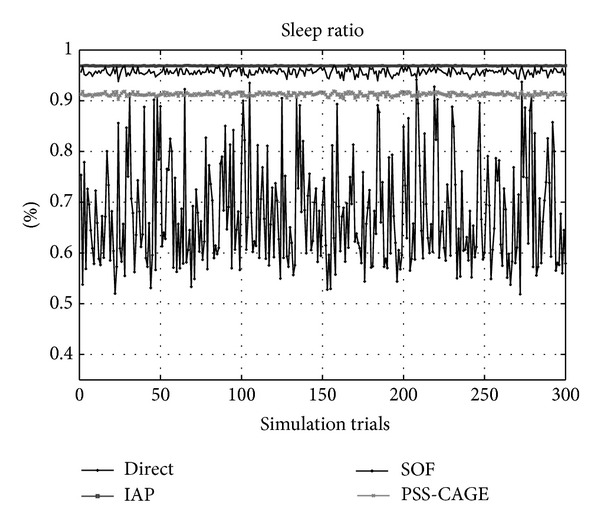
The sleep ratio (the sleep period to total simulation duration).

Figure 6 presents the sleep ratio which represents the sleep period to total simulation duration. The average sleep ratio that is achieved using the Direct method is under 65%. It is the worst method in the simulation. In this method, the traffic arrival rate completely determines the duration of the sleep cycle of an MSS. All MSSs must enter the active state whenever it receives arriving traffic. Aggregating packets and the computation of the sleep cycle enable the sleep ratio of 96.5% to be obtained by the IAP method. The sleep ratio that is achieved using the SOF method ranges from 94% to 96.5%. It is slightly worse than that achieved using the IAP method. The ratio achieved using the PSS-CAGE is approximately 92%. When an MSS cannot be scheduled, the SOF method can split the overflowing data to allocate some to a new active OFDM frame but will not shorten the original sleep cycle of the MSS. However, the PSS-CAGE will allow the MSS to use a sleep cycle with a shorter period to generate fewer data in each active OFDM frame. Therefore, SOF has a better sleeping ratio than PSS-CAGE. As presented in Figure 4, the SOF method provides the best bandwidth utilization but its sleep ratio is slightly less than that achieved using the IAP method. Whereas the IAP method sacrifices approximately 1% of the sleep ratio, it improves the bandwidth utilization by more than 10%.

**Figure 7:**
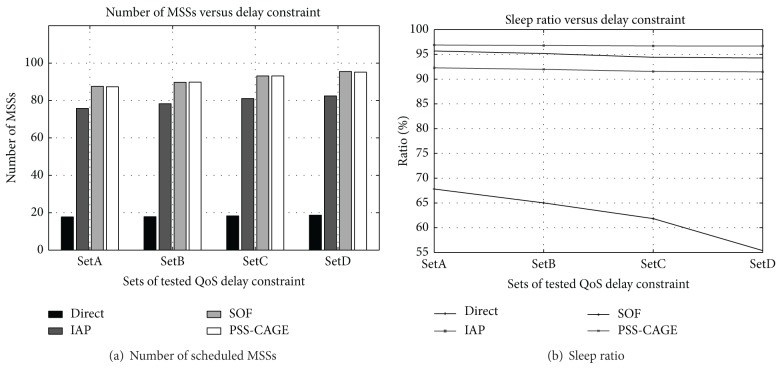
Effect of QoS time constraint on scheduled MSSs and sleep ratio.

Figure 7 plots the relationship between the sleep ratio and the QoS time constraint. In the first group, every connection of an MSS will randomly select its QoS time constraint from SetA, {150 ms, 200 ms, 250 ms, 300 ms}.  Table 1 lists the corresponding SetB, SetC, and SetD. The number of scheduled MSSs increases as the variation of the QoS time constraint among connections decreases, as displayed in Figure 7(a). A low variation of the QoS delay constraint makes avoidance of the overlapping of active OFDM frames among MSSs easy.

Figure 7(b) plots the relation between the sleep ratio and the QoS time constraint. In the IAP and SOF methods, the connection that has the strictest QoS time constraint determines the sleep ratio of an MSS. Therefore, their sleep ratios obtained using these two methods do not fluctuate very much. The sleep ratio achieved using SOF is slightly worse than that achieved using IAP but better than that of PSS-CAGE. The SOF splits the overflowing OFDM frames into additional active OFDM frames on demand, but the PSS-CAGE method shortens the sleep cycle to scatter the transfer data. Its active pattern needs more active OFDM frames, and the sleep ratio is worse than that of SOF. The lack of a data aggregation mechanism causes the Direct method to yield a poorer sleep ratio because it has a stricter QoS time constraint.

**Figure 8:**
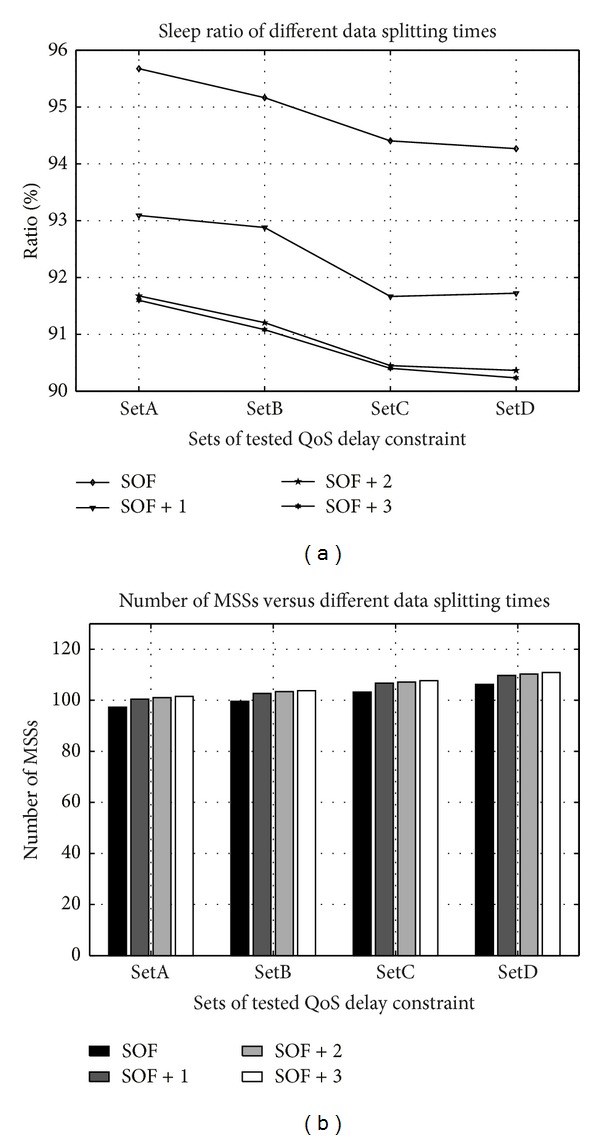
Influences of data splitting time in SOF method on scheduled MSSs and sleep ratio.

Figure 8 plots the effects of the number of data splitting times in the SOF method on the sleep ratio and the number of scheduled MSSs. The value *i* in SOF + *i* is the additional number of instances of data splitting to schedule MSSs. Performing one more data split lowers the sleep ratio by 2.5%, as displayed in Figure 8(a), while increasing the number of scheduled MSSs by only three, as displayed in Figure 8(b). Adding two data splits reduces the sleep ratio by 4% and adding three data splits reduces it by 4.05%. Increasing the splitting times to three does not significantly reduce the sleep ratio because almost all the available bandwidth for scheduling is used. Adding either two or three data splitting times increases one more scheduled MSS. Therefore, one more data splitting is selected in the SOF method.

## 5. Conclusions

This work presents two multiple MSSs power-saving scheduling methods for the downlink UGS connections of IEEE802.16e broadband wireless networks. They are IAP and SOF. In the IAP method, all MSSs interlace their active OFDM frames to retrieve their download data. The IAP method considers both bandwidth utilization and power efficiency. In the SOF method, the active pattern with fewest additional active OFDM frames is used to schedule the newly joining MSS when the IAP method cannot provide a feasible schedule. Simulation results reveal that IAP provides higher bandwidth utilization, while allowing all MSSs to retain their original sleep cycle. The SOF method improves the bandwidth utilization by more than 10%, sacrificing only 1% of the sleep ratio of the MSSs.

## Figures and Tables

**Algorithm 1:**
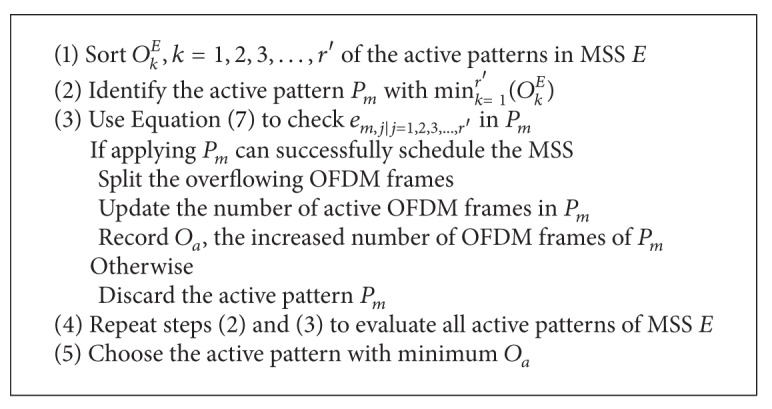
The SOF algorithm.

**Table 1:** Groups of QoS time constraints.

Group	QoS time constraints
SetA	150 ms, 200 ms, 250 ms, and 300 ms
SetB	150 ms, 200 ms, and 250 ms
SetC	150 ms, 200 ms
SetD	150 ms
